# Actual use, intention to use, and user attitudes toward mobile health applications for coronary heart disease self-management: a systematic review and meta-analysis

**DOI:** 10.1186/s12911-026-03554-6

**Published:** 2026-05-08

**Authors:** Tesema Etefa Birhanu, Elena Vlahu-Gjorgievska, Khin Than Win

**Affiliations:** 1 Department of Biomedical Sciences, Institute of Health, Jimma University, Jimma, Ethiopia; 2 Faculty of Engineering and Information Sciences, University of Wollongong, Wollongong, NSW Australia

**Keywords:** Mobile health application, Coronary heart disease, Coronary artery disease, Meta-analysis, Systematic review, Digital health system

## Abstract

**Background:**

Mobile Health applications (mHealth) have become a promising approach to support self-management of coronary heart disease (CHD). No previous studies have examined user acceptance constructs, and the results remain inconsistent. The aim of this study is to synthesize and quantify the pooled prevalence of current use, intention to use, perceived usefulness, and positive user attitudes toward mHealth apps among patients with coronary heart disease.

**Methods:**

A systematic review and meta-analysis were conducted in accordance with PRISMA guidelines and registered in PROSPERO (CRD420251018916). Five electronic databases (PubMed, Web of Science, Cochrane Library, CINAHL, and SCOPUS) were searched for studies published in English between January 2015 and April 2025. Primary studies reporting at least one acceptance-related construct: actual use, intention to use, perceived usefulness, and positive user attitude among patients with CHD or cardiac events were included. Random-effects models (metaprop, REML) were used to estimate pooled prevalence. Heterogeneity, sensitivity test, subgroup analysis, and publication bias assessments were performed.

**Results:**

A total of 6113 participants in 17 studies. The pooled prevalence of actual use was [39% (95% CI: 24%-54%)], intention to use was [61% (95% CI: 53%-69%)], perceived usefulness was [69% (95% CI: 49%-88%)], and positive user attitude was [80% (95% CI: 69%-91%)]. Substantial heterogeneity was observed across studies. Sensitivity analysis indicated no influential outliers. The funnel plot and Egger’s test indicate no statistically significant publication bias. However, the findings should be interpreted with caution due to substantial heterogeneity and a small number of studies.

**Conclusion:**

According to our findings, despite strong behavioural intention, high perceived usefulness, and positive user attitudes, actual usage remains relatively low, highlighting a gap between acceptance and implementation. The findings provide a potential quantitative basis for guiding the design and development of mHealth applications and for emphasizing user-centered interfaces to translate acceptance into sustained engagement in CHD self-management. These findings reveal significant untapped potential for mHealth-supported CHD self-care and the closely related cardiovascular population with similar self-management needs.

**Supplementary Information:**

The online version contains supplementary material available at 10.1186/s12911-026-03554-6.

## Background

Coronary heart disease (CHD) is defined as atherosclerotic narrowing or obstruction of the coronary arteries and encompasses stable angina, acute coronary syndrome (unstable angina, NSTEMI, STEMI), silent ischemia, microvascular angina, vasospastic angina, and chronic ischemic heart disease [1, 2]. CHD is the leading cause of death worldwide, responsible for approximately 8.9 million deaths and nearly 200 million DALYs, and constitutes a major part of the overall cardiovascular disease burden [3]. The lack of effective interventions in controlling CHD risk factors is the most critical factor contributing to the rising prevalence and mortality of CHD [4, 5]. The ongoing burden of CHD requires effective self-management tools, such as mobile health applications (mHealth apps), which are crucial, and understanding user acceptance is vital to their success. mHealth apps have emerged as promising tools to support ongoing monitoring and lifestyle modification, becoming a valuable means of enhancing self-management [6].

According to the Technology Acceptance Model (TAM) and the Unified Theory of Acceptance and Use of Technology (UTAUT), perceived usefulness, user attitude, intention to use, and actual use are the key sequential determinants of technology adoption. These constructs are crucial for understanding mHealth acceptance and for guiding user-centered system design and implementation strategies [7–9]. Studies regarding the connections between engagement, adherence, and self-management, as well as mHealth tool abandonment, remain inconsistent. There is no consensus on methods for measuring, quantifying, and reporting user engagement in mHealth; similarly, no agreement exists on the appropriate engagement levels required to facilitate significant behaviour change; furthermore, limited research has been conducted on user engagement as an endpoint. These critical limitations in the science of user engagement hinder the effectiveness, acceptance, and scalability of mHealth interventions aimed at promoting adherence and self-management [10, 11].

User–system interaction theory and the human-centered design framework suggest that acceptance alone isn’t enough to ensure ongoing engagement unless system interfaces, feedback mechanisms, and contextual integration are customized to users’ needs and abilities. Therefore, measuring acceptance-related constructs is essential not only for predicting initial adoption but also for providing empirical insights that inform interface development, personalization strategies, and integration into clinical workflows [12, 13]. In this context, mHealth apps offer substantial opportunities to support continuous monitoring and self-care for CHD, where ongoing engagement is critical for effective disease management [14].

Current evidence on the acceptance constructs of mHealth apps in patients with CHD remains inconsistent, and no previous systematic review has provided pooled quantitative data. No study has been conducted that specifically focuses on mHealth apps. To address these gaps, this systematic review and meta-analysis synthesizes quantitative data on acceptance-related factors, including user attitude, perceived usefulness, actual use, and intention to use, among patients with CHD or who have had cardiac events. A better understanding of acceptance constructs is crucial for policy, design, and development of effective mHealth tools that support self-management and lead to improved health outcomes.

## Methods

### Data sources and search strategies

This systematic review and meta-analysis were conducted in accordance with the Preferred Reporting Items for Systematic Reviews and Meta-Analyses (PRISMA) guidelines [15]. The protocol was registered on PROSPERO (CRD420251018916). The search was conducted across electronic databases, including PubMed, Web of Science, Cochrane Library, CINAHL, and SCOPUS, using a comprehensive search strategy tailored to each database’s requirements and incorporating Boolean operators (“OR” and “AND”).

**Fig. 1:**
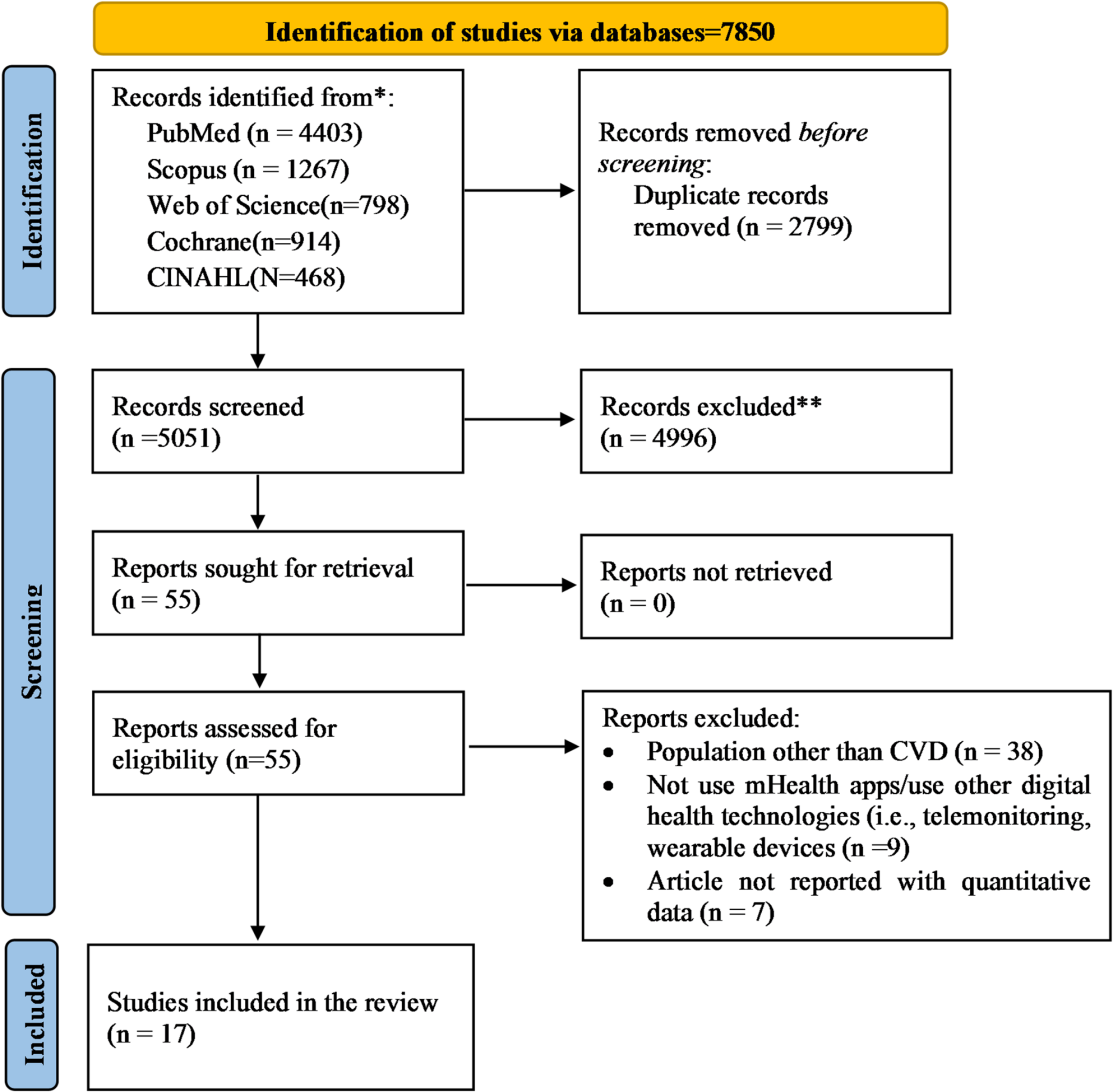
PRISMA flow diagram of study screening and selection procedures

The databases were selected to ensure comprehensive coverage of the literature across databases, and are recognized for indexing high-quality peer-reviewed studies [16, 17]. The literature search and screening were performed between 15/03/2025 and 30/05/2025. The full search strategy for each database is provided in Supplementary Data 1. A total of 7,850 research papers were identified from the databases. Based on the eligibility criteria, 4,996 articles were excluded based on the title and abstract, and 55 full-text articles were assessed for eligibility. Finally, 17 articles were included in the review. The complete flow chart of study selection is presented in Fig. 1.

### Selection of studies

All selected articles were imported into Covidence, and duplicates were removed. Titles and abstracts were screened, followed by an assessment of the full articles for eligibility. The first and second reviewers (TEB, EVG) conducted the entire screening process. Through this process, the reviewer identified relevant studies that met the inclusion criteria and aligned with the research objectives. Final decisions on each article were made collaboratively, with a third reviewer (KTW) consulted in the event of any disagreement.

### Eligibility criteria

The target population consists of adults diagnosed with CHD, including cardiac events, and also when the intervention targeted relevant self-management functions.

Primary studies were included if they met the following criteria:

- Population: Adults diagnosed with CHD, acute coronary syndrome, post-cardiac surgery patients, or those experiencing cardiac events with similar self-management needs.
- Study design: Quantitative or mixed-methods primary studies.
- Outcomes: Studies reported at least one acceptance-related construct (actual use, intention to use, perceived usefulness, user attitude).
- Language: English.
- Publication period: 2015–2025.

This review focused on CHD, but we also included individuals who were experiencing cardiac events or other closely related cardiovascular conditions. Due to the nature of the conditions, they share common self-management demands, including medication adherence, symptom monitoring, and lifestyle changes. Additionally, the acceptance-related parameters are almost similar across populations.

### Exclusion criteria

- Unpublished articles, case reports, conference papers, or dissertations.
- Studies that do not report acceptance-related outcomes.

### Quality assessment

The selected studies were thoroughly evaluated by two authors using the critical assessment method of the Joanna Briggs Institute Meta-Analysis of Statistics Assessment and Review Instrument (JBI-MAStARI) [18]. For mixed-methods studies, only the quantitative sections were extracted and assessed with the JBI Checklist, in line with JBI guidance to evaluate only the components that contribute data to the review [19]. The study employed the JBI Critical Appraisal Tools checklist, which consists of nine items with response options of “Yes,” “No,” or “Not applicable.” Four studies achieved a total score of 9 out of 9, while the remaining studies scored between 6 and 8. This method was used to evaluate the methodological rigor and overall quality of the included studies, as shown in Table 1.

**Table 1:** Methodological quality of included studies using the JBI assessment scale

Authors and year	Quality of Domain									Total score
	Q1.	Q2.	Q3.	Q4.	Q5.	Q6.	Q7.	Q8.	Q9.	
Cajita et al., 2017 [20]	Y	Y	Y	Y	Y	Y	Y	Y	N	8/9
Jiang et al., 2019 [21]	Y	Y	Y	Y	Y	Y	N	Y	Y	8/9
Ragheb et al., 2022 [22]	Y	Y	N	Y	Y	Y	Y	Y	Y	8/9
Leigh et al., 2022 [23]	Y	Y	Y	Y	Y	Y	N	Y	Y	8/9
Shan et al., 2019 [24]	Y	Y	Y	Y	Y	Y	Y	Y	Y	9/9
Ernsting et al., 2019 [25]	Y	Y	Y	Y	Y	Y	N	Y	N	7/9
Chen et al., 2022 [26]	Y	Y	N	Y	Y	Y	N	Y	N	6/9
Sohn et al., 2019 [27]	Y	Y	N	Y	Y	Y	Y	Y	Y	8/9
Reddy et al., 2018 [28]	Y	Y	N	Y	Y	Y	N	Y	Y	7/9
Tarte & Amirehsa., 2019 [29]	Y	N	N	Y	Y	Y	Y	Y	Y	7/9
Lalthanthuami et al., 2024 [30]	Y	Y	Y	Y	Y	Y	Y	Y	Y	9/9
Marcin T et al., 2021 [31]	Y	Y	Y	Y	Y	Y	Y	Y	Y	9/9
Feinberg et al., 2017 [32]	Y	N	Y	Y	Y	Y	Y	Y	Y	8/9
Baek et al., 2015 [33]	Y	Y	N	Y	Y	Y	N	Y	Y	7/9
Rahimi et al., 2015 [34]	Y	Y	Y	Y	Y	Y	Y	Y	N	8/9
Hewage et al., 2025 [35]	Y	Y	Y	Y	Y	Y	Y	Y	Y	9/9
Juan et al., 2025 [36]	Y	Y	N	Y	Y	Y	Y	Y	N	7/9
**Checklist items used** Q1. Was the sample frame appropriate address the target population? Q2. Were study participants sampled in an appropriate way? Q3. Was the sample size adequate? Q4. Were the study subjects and the setting described in detail? Q5. Was the data analysis conducted with sufficient coverage of the identified sample? Q6. Were valid methods used for the identification of the condition? Q7. Was the condition measured in a standard, reliable way for all participants? Q8. Was there appropriate statistical analysis? Q9.Was the response rate adequate, and if not, was the low response rate managed appropriately?										

Note: A scoring explanation: Y = Yes(Y = 1); N = No (*N* = 0); NA = Not Applicable

### Outcome measurement

The proportion of individuals intending to use mHealth apps for self-management was calculated as the number of interested individuals divided by the number of smartphone users, or the number of respondents to specific items. The proportion of individuals recognizing the perceived usefulness of mHealth apps was calculated as the number of people who identified mHealth apps as beneficial for enhancing self-management, lifestyle changes, and patient education, divided by the total sample size. Positive user attitude was assessed based on the proportion of participants who scored above the midpoint or an equivalent threshold on validated or adapted attitude-related items.

For each construct, we calculated the numerator (case) as the number of participants who met the study’s criteria for endorsing the construct, such as reporting app use, indicating intention to use, agreeing with the app’s usefulness, or showing a positive attitude. The denominator was defined as the number of participants who either answered the relevant items or were eligible for the specific outcome. In some studies, outcome-specific denominators differed from the overall sample size because the construct was reported only for a subsample (e.g., smartphone users, respondents to a specific acceptance item, or participants completing a designated subscale).

### Data extraction

Using a preliminary extraction form, the authors collected data on authors’ names, year of publication, study setting, study design, study population, sample size, mean age (years), primary outcomes, outcome-denominators, case/total, and response rate.

### Data synthesis

A Microsoft Excel spreadsheet was carefully filled with data sourced from relevant studies. The pertinent results were converted into an event-count format to enhance analysis. For each outcome, we extracted the exact numerator and denominator as reported in the study. In instances where outcomes were reported solely for a subsample (e.g., smartphone users, respondents to a specific acceptance item), we used the subsample denominator rather than the full study sample to avoid misclassification. Subsequently, the data were imported into STATA version 18 for analysis. Meta-analysis were performed using a random-effects model, and the restricted maximum likelihood with the metaprop function was used to estimate the pooled prevalence [37, 38]. Heterogeneity was assessed using the Cochrane chi-square (x^2^) and the index of heterogeneity (I²). Heterogeneity has been classified as low, medium, or high based on I² values of 25%, 50%, and 75%, respectively.

Subgroup analysis’s were conducted to explore potential sources of heterogeneity, including geographical location, year of publication, mean age, sample size, outcome denominators (eligibility-based vs. response-based), and study quality. Between-group differences were evaluated using the Q-test for subgroup differences [39]. Sensitivity analysis was conducted using leave-one-out analysis to assess the influence of individual studies on the pooled effect [40]. Furthermore, Publication bias was assessed using funnel plots based on log-transformed proportions and their standard errors to reduce instability associated with raw proportions. Publication bias was considered present if the p-value was < 0.10.

## Results

### Study characteristics

The study included 17 articles [20–36] with 6113 participants. The sample sizes included in the studies ranged from 35 to 1903. A total of 9 different countries were involved in the included studies: most studies, 8(47%), from the USA [20, 23, 24, 26–29, 36], 2(11.76%)from India [30, 32], and one from each: Germany [25], China [21], Canada [22], Switzerland [31], United Kingdom [34], South Korea [33], and Australia [35]. In the various articles, outcomes were assessed using adapted Technology Acceptance Model questions(TAM) [20, 34], System usability scale questions (SUS) [22], Pew Research Centre’s survey on Internet use [28], HF Management, Needs, and Acceptability of Digital Platform (HF-NADP) [30], eHealth [25] and others were adopted from reviewed articles and previous studies [21, 23, 24, 26, 27, 29, 31–33, 35, 36]. Diverse study designs, including cross-sectional and mixed methods designs, were used across the studies. The mean age of the participants ranged from 45 ± 17.18 to 77 ± 13.33 years. (Refer to Table 2).

**Table 2:** Study characteristics included

Author and year	Country	Mean Age (years)	Design	Sample size	Population	Outcome	Outcome denominators	Total	Case	Prevalence(%)	Response rate(%)
Cajita et al., 2017 [20]	USA	71.3 ± 4.6	Quantitative	129	HF	Intention to use	A smartphone user	129	55	54.5	100%
Jiang et al., 2019 [21]	China	61.07 ± 15.62	Quantitative	231	CHD, HF, or Arrhythmia	Intention to use	Respondents to outcome items	231	157	68	100%
Ragheb et al., 2022 [22]	Canada	64.3 ± 9	Quantitative	65	CABG	Intention to use	A smartphone user	46	31	67.4	100%
Leigh et al., 2022 [23]	USA	61.3 ± 12.5	Quantitative	144	CHF	mHealth utilization	A smartphone user	68	15	22	69.4%
Shan et al., 2019 [24]	USA	61.74 ± 12.25	Quantitative	1903	CVD	mHealth utilization	A smartphone user	1409	547	43	100%
Ernsting et al., 2019 [25]	Germany	55.47 ± 8	Quantitative	1334	MI, CAD	mHealth utilization	A smartphone user	1309	386	29.49	100%
Chen et al., 2022 [26]	USA	68 ± 13	Quantitative	100	ACS	Intention to use	Respondents to outcome items	100	65	65	100%
Sohn et al., 2019 [27]	USA	64.5 ± 8.3	Quantitative	95	HF	Intention to use	A smartphone user	50	30	60	47.5%
Reddy et al., 2018 [28]	USA	57.92 ± 14.17	Quantitative	306	CVD	mHealth utilization	A smartphone user	290	102	35	94.7%
Tarte and Amirehsa, 2019 [29]	USA	69.24 ± 10.4	Quantitative	48	HF	Intention to use	A smartphone user	37	26	70.3	77%
Lalthanthuami et al.,2024 [30]	India	55.54 ± 10.33	Quantitative	90	HF	Intention to use	A smartphone user	70	57	81.4	100%
Marcin T et al., 2021 [31]	Switzerland	59.6 ± 11.94	Quantitative	1025	CHD, CHF	Intention to use	Respondents to outcome items	757	363	48	50.2%
Feinberg et al., 2016 [32]	India	45 ± 17.18	Quantitative	297	CVD	User attitude	A smartphone user	262	242	92	92.9%
Baek et al., 2018 [33]	South Korea	49.54 ± 18.99	Mixed	35	CHD, CHD	Intention to use	A smartphone user	35	33	94	100%
Rahimi et al., 2015 [34]	UK	77 ± 13.33	Mixed	58	HF	mHealth utilization	Respondents to outcome items	52	33	63	89.6%
Hewage et al., 2025 [35]	Australia	50.5 ± 18.2	Quantitative	302	CHD	Intention to use	A smartphone user	302	136	45	100%
Juan et al., 2025 [36]	USA	60.17 ± 12.42	Mixed	55	CHD, PAD	Intention to use	Respondents to outcome items	37	33	89	67%

Note: HF: Heart failure. ACS: Acute coronary syndrome. CVD: Cardiovascular disease. CHD: Coronary heart disease. CHD: Chronic heart disease. IHD: Ischemic heart disease. CAD: Coronary artery disease. PAD: Peripheral artery disease. CHF: Chronic heart failure. MI: Myocardial infarction. CABG: Coronary artery bypass graft. CHF: Chronic heart failure

*Note*: Outcome denominators are the number of participants included in the analysis for each outcome and may differ from the total sample size. In some studies, estimates were restricted to smartphone users, while others included both users and non-users, or only participants who responded to outcome items or subscales

Cardiac event conditions were included because they have similar self-management needs and application functions

### Future intention to use mHealth apps for CHD self-management

**Fig. 2:**
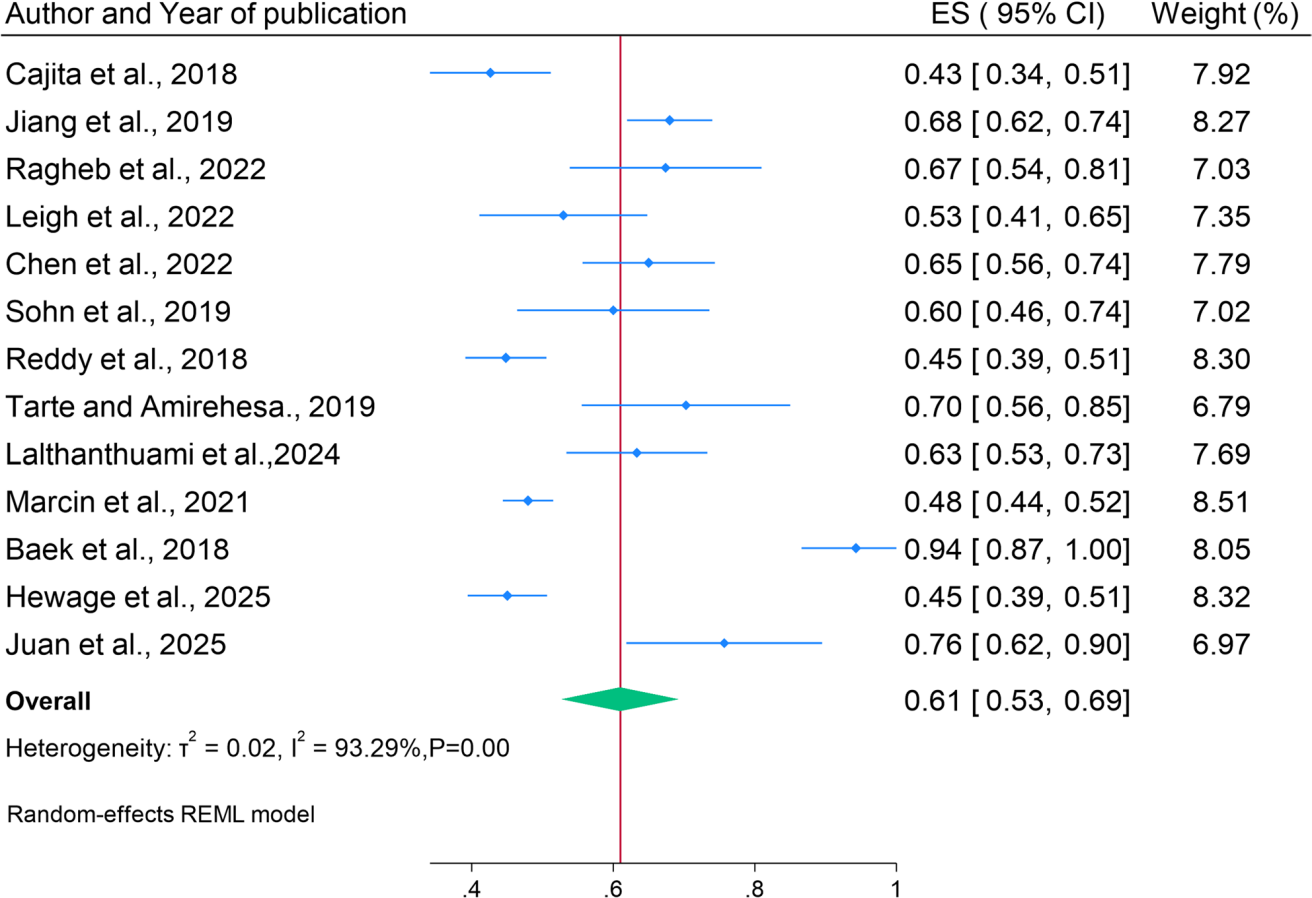
The pooled prevalence of future intention to use mHealth apps for CHD self-management

A total of thirteen [20–23, 26–31, 33, 35, 36] articles reported on the future intention to use mHealth apps for CHD self-management. The proportion of individuals intending to use mHealth apps for self-care ranges from 43% to 94%. According to metaprop, the pooled prevalence of future intention to use mHealth apps for CHD self-management was 61% (95% CI: 53%-69%) (Fig. 2).

### Actual use of mHealth apps for CHD self-management

**Fig. 3:**
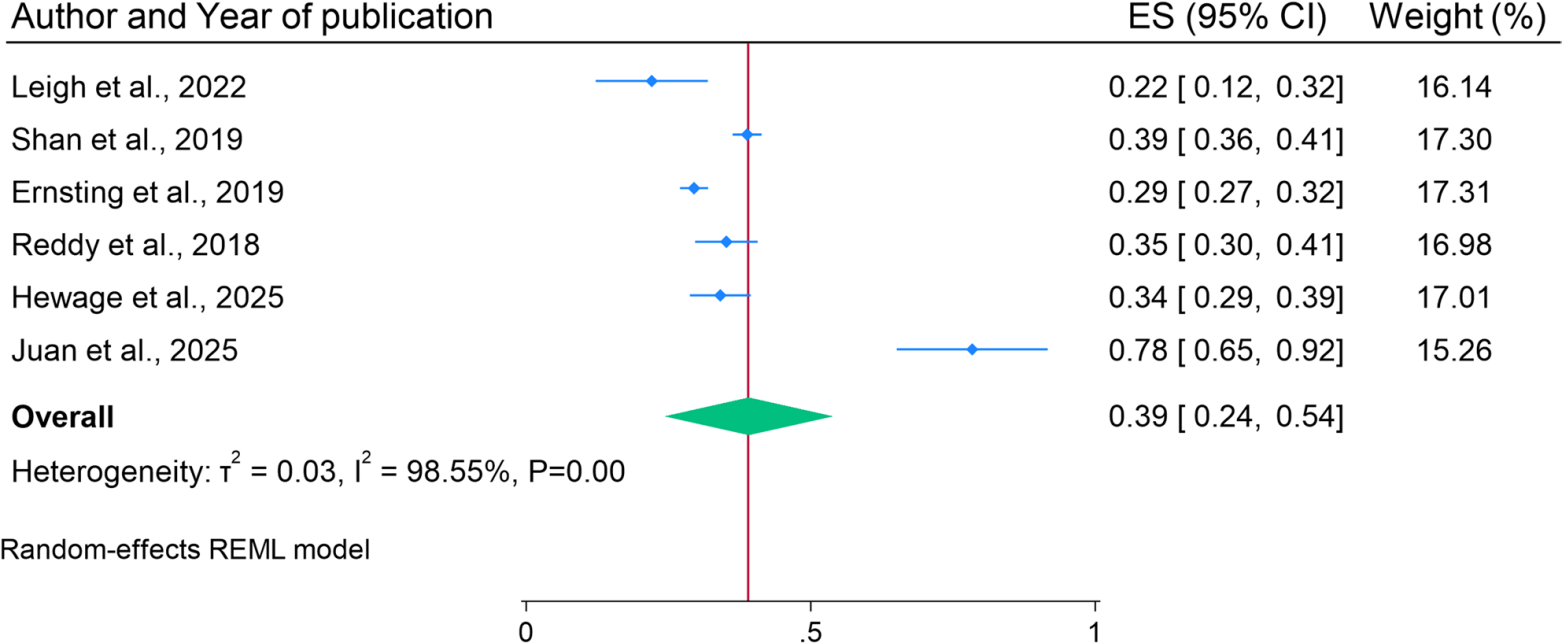
The pooled prevalence of current mHealth app usage for CHD self-management

Seven articles [22–25, 28, 35, 36] reported on the current usage of mHealth apps for CHD self-management. The reviewed papers showed that the proportion of individuals using mHealth apps ranged from 22% to 78%. Based on a metaprop, the pooled prevalence of current mHealth app use for CHD self-management was 39% (95% CI: 24%-54%) (Fig. 3).

### Perceived usefulness of mHealth apps for CHD self-management

**Fig. 4:**
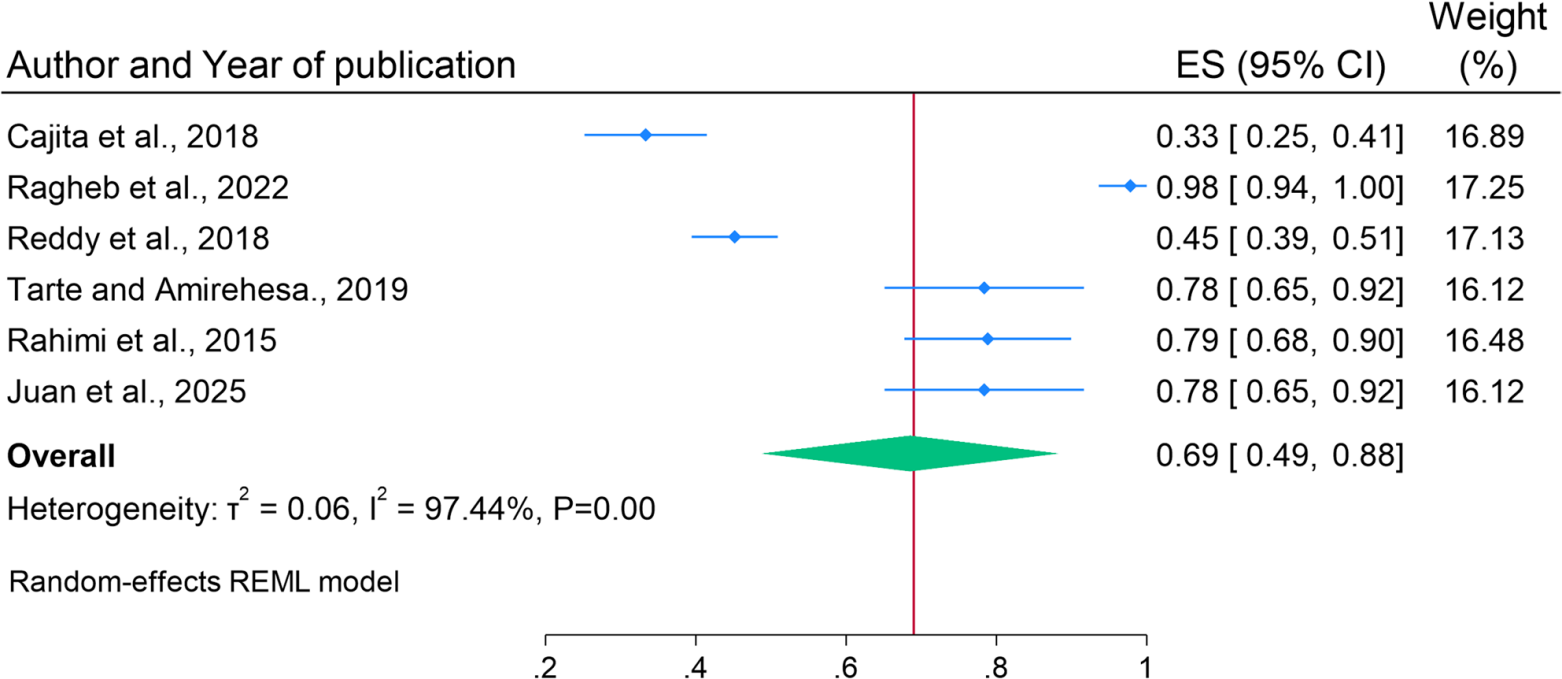
The pooled prevalence of the perceived usefulness of mHealth apps in patients with CHD

Six articles [20, 22, 28, 29, 34, 36] were reported on the perceived usefulness of mHealth apps in self-care management. The proportion of individuals ranged from 33% to 98%. The overall pooled estimate of the perceived usefulness of mHealth apps was 69% (95% CI: 49%-88%) (Fig. 4).

### Positive user attitude of mHealth apps for CHD self-management

**Fig. 5:**
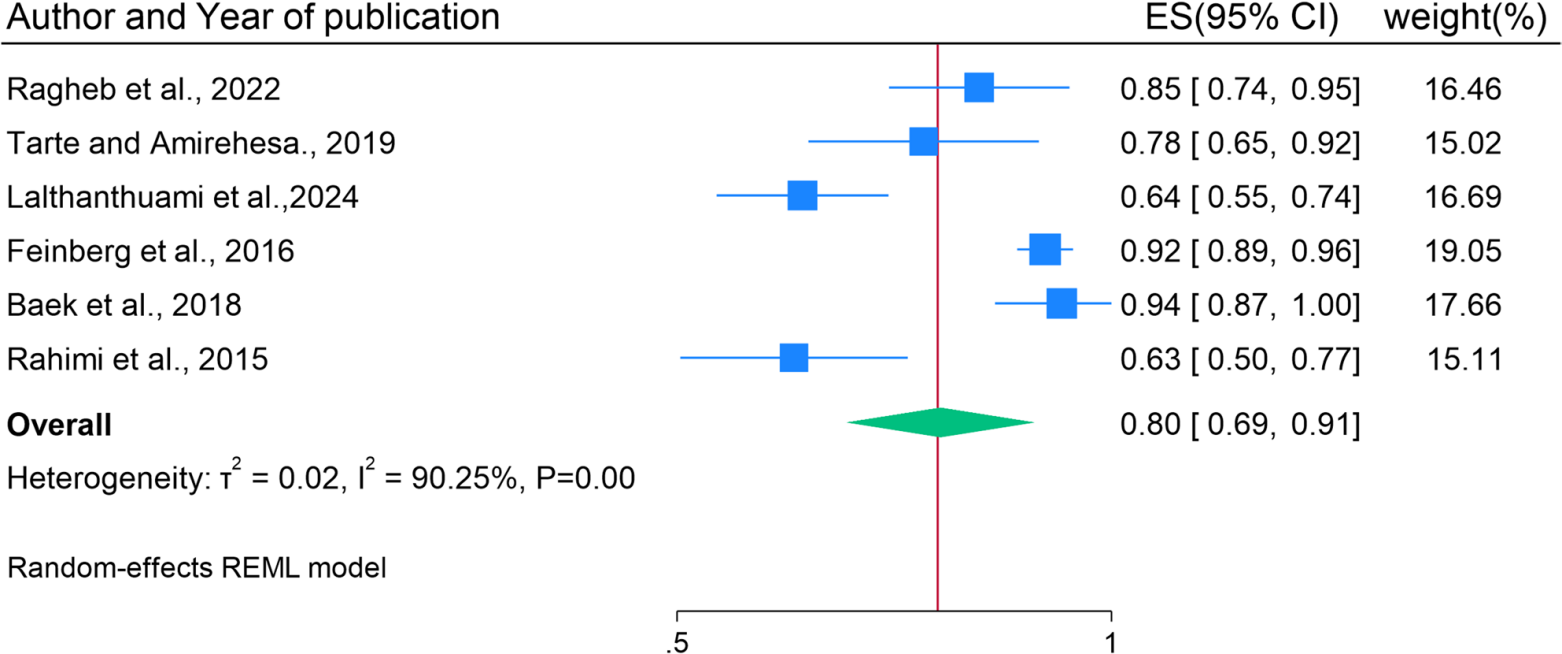
The pooled prevalence of positive user attitudes toward mHealth apps in patients with CHD

Six articles [22, 29, 30, 32–34] were reported regarding positive users’ attitudes toward mHealth apps for self-management. The proportion of individuals ranges from 63% to 94%. The overall pooled estimate of positive user attitude towards mHealth apps for CHD self-management was 80% (95% CI: 69%-91%) (Fig. 5).

### Publication bias

**Fig. 6:**
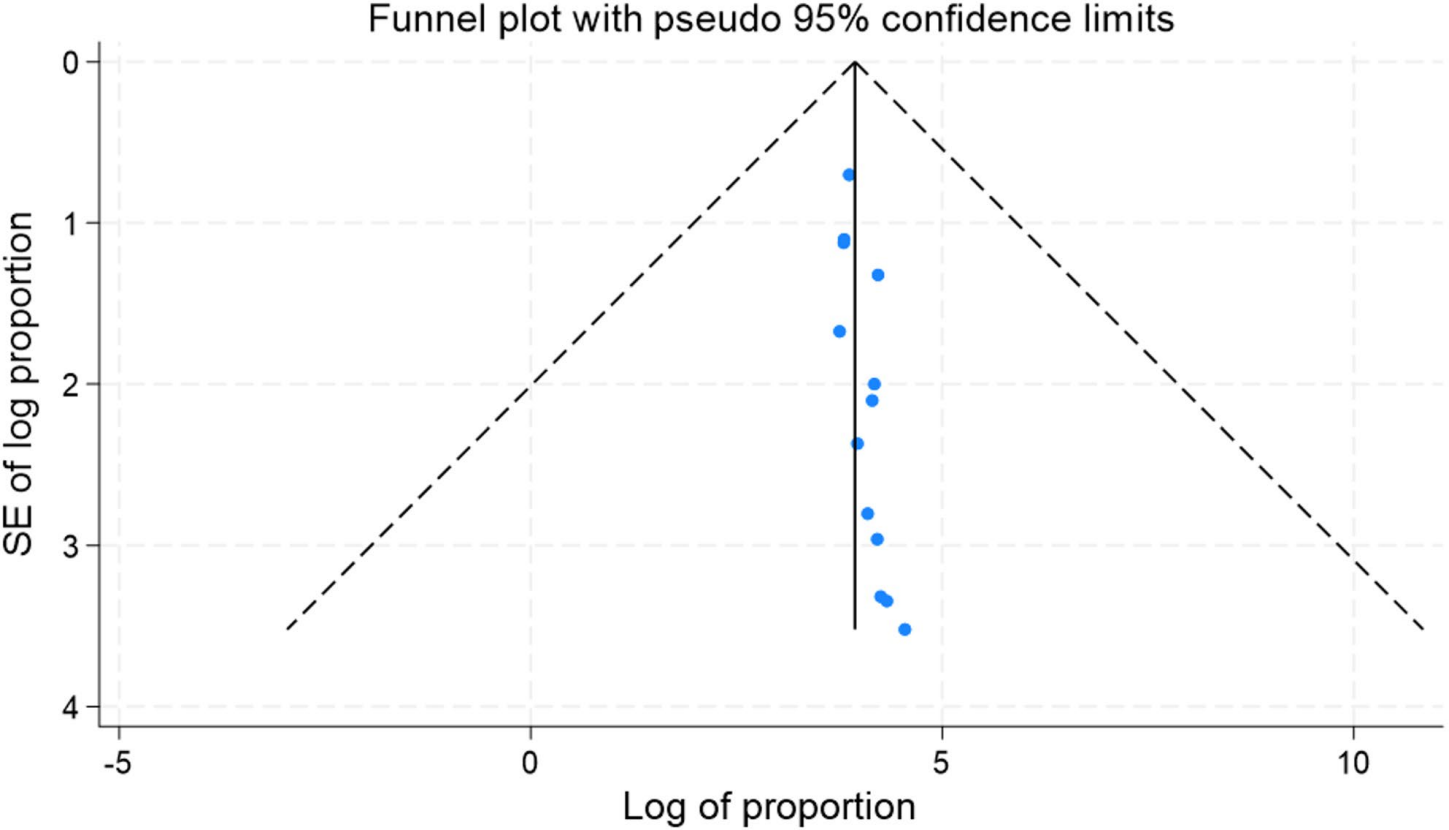
Funnel plot for assessing publication bias of future intention to use mHealth apps for CHD self-care

**Fig. 7:**
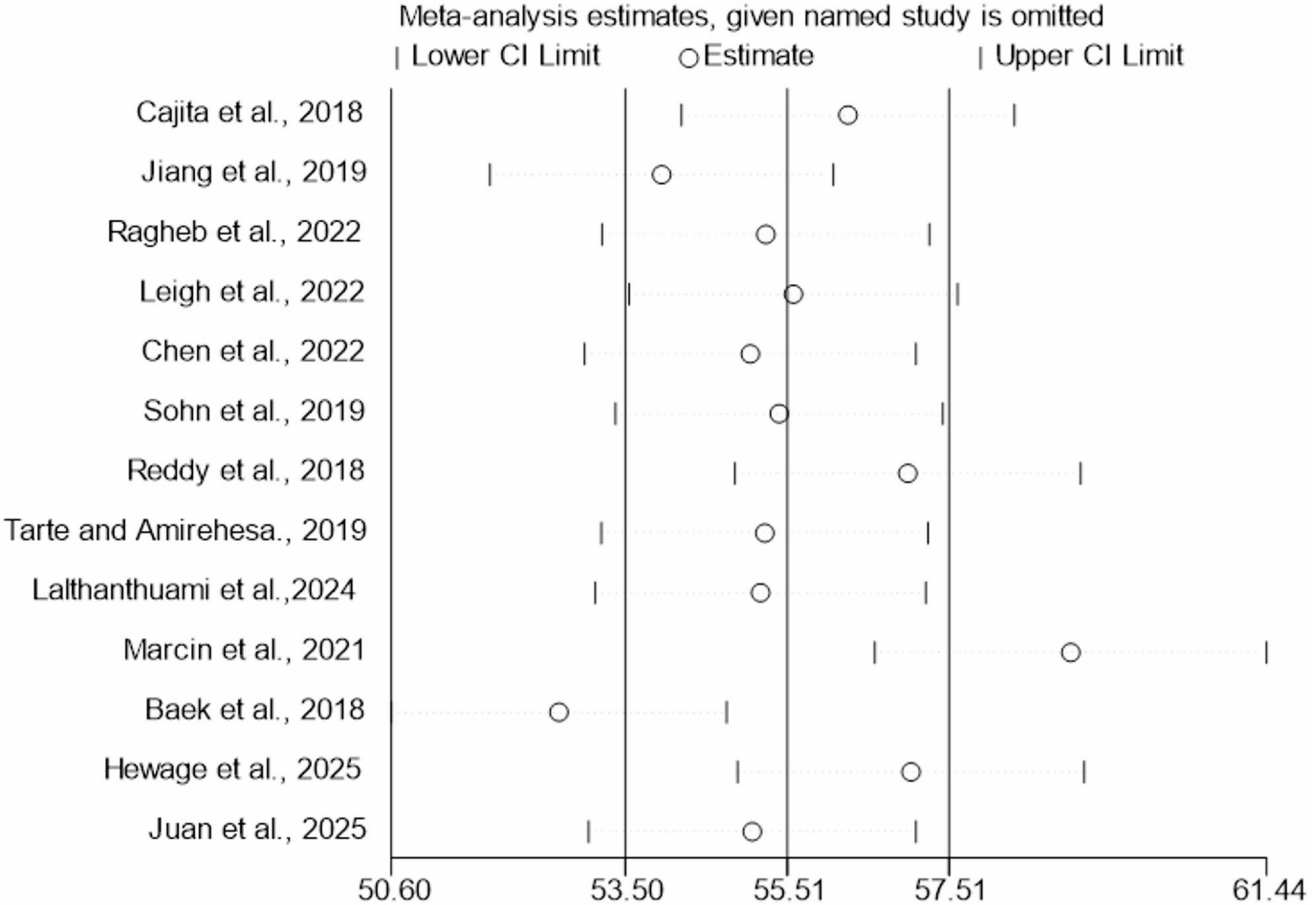
Leave-one-out sensitivity analysis of the pooled prevalence estimate

The funnel plot shows no visual indication of directional asymmetry, indicating a symmetrical distribution of study estimates around the pooled effect size. However, no definitive conclusions about publication bias can be drawn because some pooled outcomes included fewer studies and exhibited significant heterogeneity (Fig. 6). A Sensitivity analysis was conducted using a leave-one-out approach and by excluding studies in which non-CHD cardiovascular patients were the main sample to evaluate the robustness of the results. According to the results, all point estimates were within the 95% Confidence Interval (Fig. 7).

### Random- effects meta-regression results

The random-effects meta-regression model accounted for 37.5% of the heterogeneity between studies (R² = 37.51%) and was statistically significant overall (Wald χ²(4) = 10.23, *p* = 0.0367). None of the individual covariates—specifically, mean age (*p* = 0.576), sample size (*p* = 0.128), study quality (*p* = 0.358), or year of publication (*p* = 0.559)—were statistically significant predictors of acceptance results. A significant residual heterogeneity remained (I²=88.6%), indicating that an unmeasured factor likely contributed to variability across the experiments(as shown in Table 3).

**Table 3:** Random-effects meta-regression results

Covariate	Coefficient (β)	Std. Error	z‑value	*p*‑value	95%CI
Mean age	5.53	9.89	0.56	0.576	-13.84-24.91
Sample size	-14.91	9.81	-1.52	0.128	-34.12-4.30
Study quality	-9.31	10.12	-0.92	0.358	-29.15-10.53
Year of publication	5.16	8.83	0.58	0.559	-12.14-22.45
Intercept	81.7	28.96	2.80	0.005	24.41-137.92

Note: I² (%) = 88.61%, R² (heterogeneity explained) = 37.1%, Wald χ² (df = 4) = 10.23, p‑value = 0.0367

### Handling heterogeneity

**Fig. 8:**
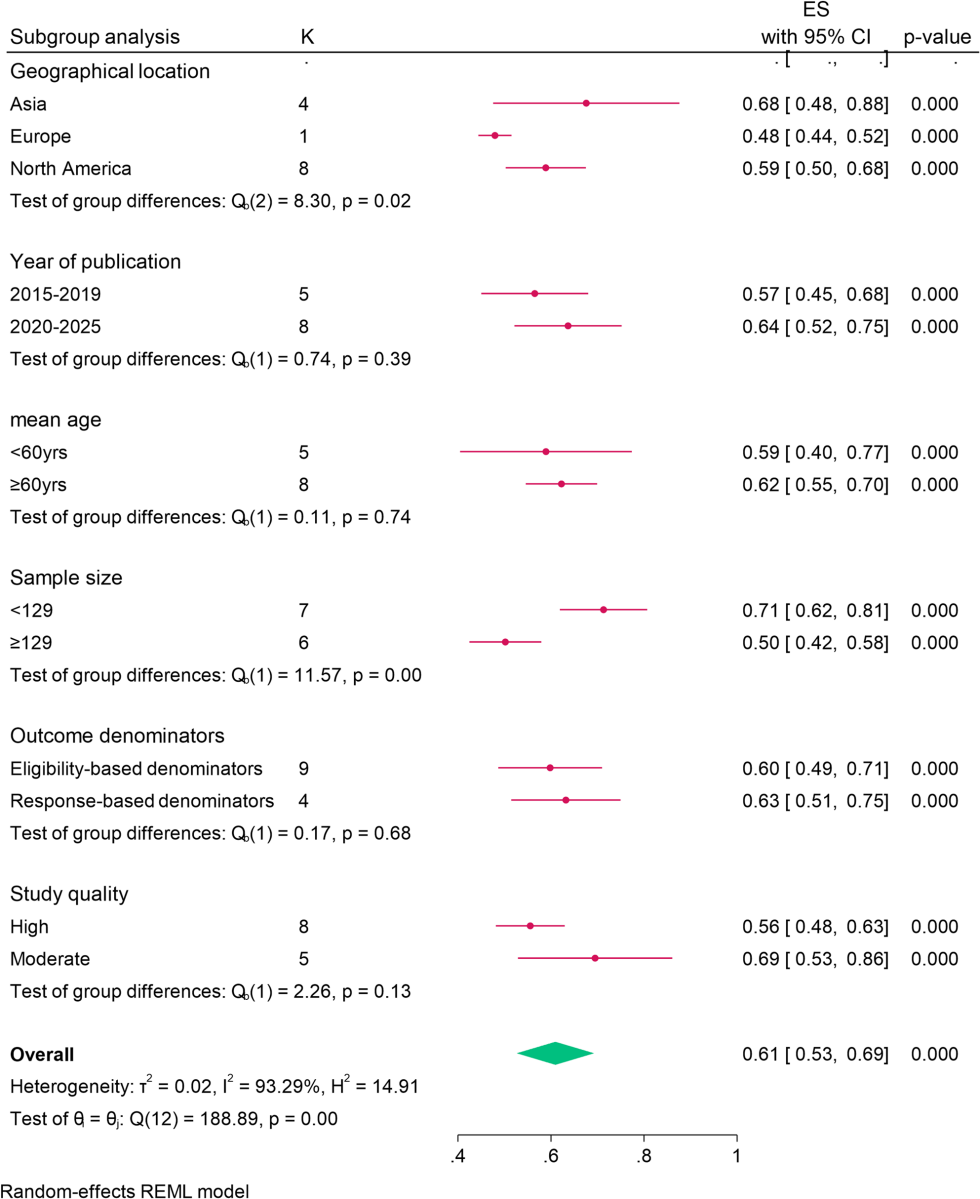
Forest plot illustrates subgroup analysis of future intent to utilize mHealth applications for self-management of coronary heart disease

The pooled estimate from the random-effects model showed substantial heterogeneity. Subgroup analyses were performed to ascertain the source of this heterogeneity, considering factors such as study setting, mean age (years), sample size, publication year, study quality, and outcome denominators (see Fig. 8). Geographical location and sample size are the only moderators that significantly explain the heterogeneity between studies. Differences in region-specific contexts and small-study effects likely contribute to the high heterogeneity. However, age, year of publication, denominator type, and study quality do not appear for the observed variability. No statistically significant difference was observed between studies utilizing eligibility-based denominators (e.g., smartphone users) and those employing response-based denominators (e.g., respondents to items). This indicates that changes in analytic denominators did not significantly influence the pooled estimates.

## Discussion

This systematic review and meta-analysis synthesized quantitative evidence on actual use, intention to use, perceived usefulness, and positive user attitude towards mHealth applications among patients with coronary heart disease and closely related cardiovascular conditions. According to the pooled findings, perceived usefulness (69%) and positive user attitudes (80%) toward mHealth apps were high. Within the TAM, these cognitive and affective evaluations are central determinants of behavioral intention, which was also moderately high in our analysis(61%). However, actual usage remained relatively low (39%), indicating that intention does not always lead to sustained engagement. Additionally, this finding suggests that more than 60% of the participants did not use mHealth applications for self-management. Studies have identified factors associated with low engagement in mHealth apps, including low digital literacy, symptom burden, usability issues, disease-related fatigue, and low digital confidence [11, 41, 42]. This finding is also consistent with the UTAUT framework, which identifies perceived accuracy, perceived privacy protection, and perceived convenience as central constructs that influence both behavioral intention and actual use [43, 44].

The discrepancy between intention to use and actual use in this finding may highlight the intention-behavior gap, in which individuals express a desire to adopt a technology but fail to translate that intention into sustained action. This discrepancy is frequently attributed to external and contextual obstacles, such as inadequate integration into clinical workflows and the absence of continuous feedback mechanisms [45, 46]. And also, previous studies indicated that factors like limited clinical feedback loops, a high manual data-entry burden, and a lack of system integration can contribute to the intention-behavioral gap [47, 48].

The pooled prevalence of future intention to use mHealth apps for CHD self-management was 61% (95% CI, 53%- 69%). The evidence from included studies suggests that future interest in using mHealth apps for CHD self-management depends on preference-aligned features, a desire for better self-management and symptom control, and motivation to improve health outcomes [26, 27, 36]. This finding is also consistent with a study by Xie et al. [46], who reported that mHealth app acceptability increases when the app offers simple health data interpretation, is user-friendly, is supported by healthcare providers, and provides high-quality health data. These patterns also align with TAM and UTAUT studies, indicating that technology-related attributes such as compatibility, privacy protection, usefulness, convenience, and accuracy significantly influence users’ intention to adopt new technologies [49, 50]. The findings may provide approximate reference points rather than prescriptive guidance for the design and development of mHealth apps. And also underscore the importance of addressing implementation and contextual barriers alongside improvements to application functionality.

This pattern indicates a design-level engagement gap in addition to a behavioral intention [45, 51]. Nevertheless, a high acceptance score indicates a positive perception of the interventions; acceptance alone, however, does not necessarily imply adoption and engagement, especially in populations with diverse digital literacy levels and comorbidities [45, 52]. These findings are consistent with some studies that have identified factors associated with low acceptance, including limited system integration, the need for high digital literacy, lack of awareness, the absence of patient-physician communication, and the absence of physician feedback loops [21, 23, 24, 36]. Future mHealth solutions for CHD prioritize integration with remote monitoring devices, automated medication reconciliation, and compatibility with clinical workflows.

The pooled prevalence of perceived usefulness of mHealth apps for CHD self-care was 69% (95% CI: 49%-88%). This result consists of studies by Sohn et al., Baek et al., Ernsting et al., and Shan et al. [24, 25, 27, 33]. The perceived usefulness of mHealth apps depends on behavioural change techniques (such as goal setting and feedback), symptom tracking, medication reminders, integration with electronic health records, clinician feedback, automated medication import, and tailored education and personalized recommendations. Perceived usefulness is one of the strongest predictors of adoption. This finding is also consistent with human–computer interaction principles, which emphasize that a system’s efficacy depends on the alignment of user requirements, interface design, and integration capabilities [53]. This also aligns with the findings of Ortega EC et al. [54], who highlight that mHealth apps for self-care management are effective when developed with a user-centered approach and continuous validation, thereby enhancing treatment adherence and communication between patients and healthcare professionals.

The pooled prevalence of positive user attitudes towards mHealth apps for CHD self-management was 80% (95% CI: 69%-91%). Although there is a lack of comparable studies for comparison, this finding suggests that nearly 20% of the research participants did not have a positive user attitude towards mHealth applications. Studies have identified several hindering factors, including language barriers, restrictive religious beliefs, concerns about the accuracy of the information provided, privacy concerns, transparency in data use, individual experience, confidentiality, and lack of knowledge [33, 55, 56]. Furthermore, barriers include slow performance, poor integration with electronic health records, clinical time constraints, and a gap between user interest and practical application [26, 27, 33, 36]. These findings highlight that a positive user attitude is important but insufficient for adoption, underscoring the need to address other barriers beyond user perception. Overcoming broader technical, contextual, and trust-related barriers remains essential for effective mHealth uptake.

The pooled prevalence estimates may be interpreted with caution. Because intention, usefulness, and attitude are latent constructs measured using different scales, cut-offs,2 and denominators. The pooled estimates showed substantial heterogeneity. Although subgroup analysis were conducted to identify potential sources of variability, heterogeneity remained significant across most categories. As a result, these subgroup patterns should be viewed as exploratory indicators rather than explanatory factors of acceptance. This diversity may be due to differences in study demographics, outcome-measuring methodologies, mHealth features, and healthcare environments. Although the meta-regression model accounted for 37.5% of the variability, none of the study-level factors predicted acceptance outcomes independently. This suggests that variables not captured in the published research characteristics, such as variations in app functionality, patients’ digital literacy, clinical settings, or cultural influences, affect variability across studies. The persistent high residual heterogeneity highlights the complexity of mHealth acceptability and the need for more consistent measurement methods in future research.

The findings of this review indicate a significant gap between patients’ positive perceptions of mHealth applications and their actual use. This difference has significant implications for the design, implementation, and administration of digital health systems employed in CHD self-management and other closely related cardiovascular conditions. The finding highlights the need to integrate mHealth tools into standard CHD self-management practices at the health system level. Healthcare systems are encouraged to develop and adopt interoperability standards for electronic health records to enable doctors to access patient-generated data and respond promptly.

### Strengths and limitations of the study

This systematic review and meta-analysis possess several notable strengths. The study was carried out in accordance with PRISMA guidelines and was prospectively registered with PROSPERO. The study selection and screening were performed using the Covidence database management software. The JBI critical appraisal tools were used to assess methodological quality, and pooled prevalence estimates were calculated using random-effects models. Sensitivity, subgroup, and publication bias analyses were performed to enhance the reliability of the results.

There are some limitations. The pooled estimates demonstrated substantial heterogeneity. In the subgroup analysis, geographical location and sample size were identified as contributing to heterogeneity; however, a substantial portion of the heterogeneity remained unexplained. These factors may be due to differences in study design, digital literacy level, demographics, and measurement instruments. Despite discrepancies in assessment tools across studies, all outcomes reflected participants’ views on acceptance, which also contributes to heterogeneity. Outcome denominators vary across studies because some estimates were based on smartphone users or respondents to specific items rather than the entire sample. Given the substantial heterogeneity, differences in outcome definitions, and the inclusion of the cardiovascular population, the findings should be interpreted as suggestive rather than definitive, offering a general overview of acceptance patterns rather than specific design guidelines. Some studies included broader cardiovascular populations, which might reduce the generalizability of the findings to patients with CHD alone. Additionally, most primary studies were conducted in high-income nations, thereby limiting the applicability of the findings to the global population.

## Conclusion

The finding highlights general patterns of acceptance among individuals with CHD and closely related cardiovascular conditions. Around two-thirds of patients with CHD expressed an intention to use mHealth apps for CHD self-management. These findings suggest an approximate range for understanding how patients with CHD and closely related cardiovascular conditions accept and use mHealth applications. However, due to significant heterogeneity, differences in measurement methods, and the inclusion of a broader cardiovascular population, these findings may be interpreted with caution. They underscore a general pattern that may inform future research on longitudinal, cohort, and integrated clinical studies aimed at examining how perceived usefulness and positive user attitudes influence sustained use of mHealth applications, especially in underrepresented middle- and low-income settings.

## Electronic Supplementary Material

Below is the link to the electronic supplementary material.

Supplementary Material 1

Supplementary Material 2

Supplementary Material 3

## Data Availability

No new primary data were collected; all data analyzed come from previously published studies cited in this manuscript.

## Abbreviations

CHD: Coronary Heart Disease
CVD: Cardiovascular Disease
ES: Effect Size
JBI: Joanna Briggs Institute
mHealth apps: Mobile Health Application
PRISMA: Preferred Reporting Items for Systematic Reviews and Meta-Analyses
TAM: Technology Acceptance Model
USA: United States of America
UTAUT: The Unified Theory of Acceptance and Use of Technology
