# Supplementary material for: Actual use, intention to use, and user attitudes toward mobile health applications for coronary heart disease self-management: a systematic review and meta-analysis
Source: BMC Med Inform Decis Mak. 2026 May 8;26:232. doi: 10.1186/s12911-026-03554-6 (PMC13321549; doi:10.1186/s12911-026-03554-6)
Supplement: Supplementary file 2 — Supplementary Material 2 [file 12911_2026_3554_MOESM2_ESM.docx]

**Supplementary Materials**

**Supplementary data 1**: Database search keywords for each database

| **Databases=**  7850 | **Search** | **Search terms** |
| --- | --- | --- |
| PubMed  4403 | #1 | (("cardiovascular disease") OR ("cardiac disease") OR ("heart disease") OR ("coronary heart disease*") OR ("coronary artery disease*") OR ("myocardial infarction") OR ("myocardial ischemia") OR ("acute coronary syndrome*") OR ("heart failure") OR ("Arrhythmia") OR ("Valvular heart disease") OR ("congenital heart disease") OR ("cardiomyopathy")) |
|  | #2 | (("mobile health applications") OR ("mHealth apps") OR ("mHealth") OR ("telehealth") OR ("telemedicine") OR ("Smartphone apps") OR ("mobile apps") OR ("apps") OR ("computer-based")) |
|  | #3 | ("perceived usefulness*") OR ("perceived ease of use") OR (perception) OR ("attitude*") OR ("usage*") OR ("user experience") OR ("intention to use*") OR ("satisfaction") OR ("self-care") OR ("self-management") |
| WOS  798 | #1 | TS= (cardiovascular disease OR cardiac disease OR heart disease OR coronary heart disease* OR coronary artery disease* OR myocardial infarction OR myocardial ischemia OR acute coronary syndrome OR heart failure OR Arrhythmia OR Valvular heart disease OR congenital heart disease OR cardiomyopathy) |
|  | #2 | TS= (mobile health applications OR mHealth apps OR mHealth OR telehealth OR telemedicine OR Smartphone apps OR mobile apps OR apps OR computer-based) |
|  | #3 | TS= (perceived usefulness* OR perceived ease of use OR perception OR attitude* OR usage* OR user experience OR intention to use* OR satisfaction OR self-care OR self-management) |
| Scopus  1267 | #1 | ("cardiovascular disease" OR "cardiac disease" OR "heart disease" OR "coronary heart disease" OR "coronary artery disease" OR "myocardial infarction" OR "myocardial ischemia" OR "acute coronary syndrome" OR "heart failure" OR "arrhythmia") |
|  | #2 | ("Mobile health application" OR "mHealth apps" OR "mHealth" OR "telehealth" OR "telemedicine" OR "smartphone apps" OR "mobile apps" OR "apps" OR "computer-based") |
|  | #3 | ("Perceived usefulness*" OR "perception" OR "attitude*" OR "usage*" OR "user experience" OR "satisfaction" OR "intention to use*" OR "self-care" OR "self-management") |
| Cochrane  914 | #1 | (cardiovascular disease OR cardiac disease OR heart disease OR coronary heart disease OR coronary artery disease OR myocardial infarction OR myocardial ischemia OR acute coronary syndrome OR heart failure OR Arrhythmia OR Valvular heart disease OR congenital heart disease OR cardiomyopathy) |
|  | #2 | (mobile health applications OR mHealth apps OR mHealth OR telehealth OR telemedicine OR Smartphone apps OR mobile apps OR apps OR computer-based) |
|  | #3 | (perceived usefulness OR perceived ease of use OR perception OR attitude OR usage OR user experience OR intention to use OR satisfaction OR self-care OR self-management) |
| CINAHL  468 | #1 | (cardiovascular disease OR cardiac disease OR heart disease OR coronary heart disease OR coronary artery disease OR myocardial infarction OR myocardial ischemia OR acute coronary syndrome OR heart failure OR Arrhythmia OR Valvular heart disease OR congenital heart disease OR cardiomyopathy) |
|  | #2 | (mobile health applications OR mHealth apps OR mHealth OR telehealth OR telemedicine OR Smartphone apps OR mobile apps OR apps OR computer-based) |
|  | #3 | (perceived usefulness OR perceived ease of use OR perception OR attitude OR usage OR user experience OR intention to use OR satisfaction OR self-care OR self-management) |
